# Experiences of Community Doulas Working with Low-Income, African American Mothers

**DOI:** 10.1089/heq.2018.0045

**Published:** 2019-04-08

**Authors:** Kristina Wint, Thistle I. Elias, Gabriella Mendez, Dara D. Mendez, Tiffany L. Gary-Webb

**Affiliations:** ^1^ Department of Behavioral and Community Health Sciences, Pittsburgh, Pennsylvania.; ^2^ Department of Epidemiology at the University of Pittsburgh Graduate School of Public Health in Pittsburgh, Pittsburgh, Pennsylvania.

**Keywords:** African American, doulas, health equity, pregnancy, qualitative research, SES

## Abstract

**Purpose:** The aim of this study was to learn from doulas the components of their services that might best serve low-income, African American (AA) women and to show the significance of doulas in helping these women have healthy, positive, birth experiences.

**Methods:** Ten doulas were recruited from a local community doula program and through word-of-mouth referrals from participants and completed in-depth interviews. Interviews were transcribed verbatim and analyzed using Atlas.ti software to identify emerging themes. Thematic saturation was achieved in interviews.

**Results:** Several themes emerged from the interviews including: (1) The influence of similarities of race, culture, and lived experience on doula care; (2) How doulas often provide birthing persons with support and resources beyond birth; and (3) How doulas recognize the institutional biases that exist in the health care system and try to mediate their effect on birthing persons.

**Conclusions:** These themes highlight how doulas can support birthing persons to mitigate the negative effects of social determinants of health, specifically racism and classism, and highlight potential avenues for doulas to consider when working with birthing persons who have low income and are AA.

## Introduction

The use of doulas, nonclinical support paraprofessionals, who provide physical, informational, and emotional support has been shown to have positive results on birth-specific and postpartum-specific outcomes.^1,2^ Doula services are associated with decreased cesarean rates, higher Apgar scores, shorter labors, and increases in breastfeeding initiation and retention.^3^ In addition, an argument has been made positioning doulas as disrupters of negative experiences while interfacing with the health care system.^3^ For example, doulas help birthing individuals navigate the health care system, given the complexities of the system as well as the experience of discrimination many African Americans (AA) face when interacting with the system.^4–7^

Despite providing a host of potential benefits, doulas often go unknown and underutilized.^8^ It is estimated that 40% or more of women are unaware of doula care and the potential support doulas can provide.^8^ In addition, cost is the greatest barrier to doula services, with doula support traditionally limited to birthing persons with the financial means to hire them.^8,9^ As a result, doula care has come to be viewed as a privilege reserved for wealthy, white people capable of paying for the resource. Despite this, low-income AA women often report wanting this support during labor and delivery.^8^

Community doula programs work to fill these voids, providing doula support for free, or reduced cost. Although traditional doulas provide support only during birth, community doula programs include traditional birth support, while adding prenatal and, at times, postnatal home-visiting services.^10,11^ In these instances, community doulas facilitate prenatal education, postnatal check-ins, and emotional support throughout pregnancy.^10,11^ Moreover, in the community doula model, doulas often come from the same community as the birthing persons receiving their services.^10,11^

Previous published reports and studies on community doula programs have explored how doulas' help positively involve partners and how they promote the initiation and retention of breastfeeding.^3,10,11^ Recent qualitative studies have explored the motivations of women of color entering the doula profession, whereas another qualitative study of community doulas and their work with immigrant women in Washington state explored the influences of cultural beliefs on doulas' ability to provide support and advocate for these individuals, both of which found that culture played a large part in both doulas' path to and practice of the profession.^11–13^ Another study explored how doulas are in a unique position to witness disrespect of patients by providers during birth.^14^ In addition, there have been recent explorations of programs that train incarcerated women as doulas to help reduce recidivism.^15,16^ There is also a growing body of work exploring the experiences and outcomes associated with abortions and bereavement doulas.^17–23^ However, few studies have explored doulas' experiences working specifically with low-income or AA women.

The aims of this research study were to understand how community doulas work and interact with low-income AA women, and to explore the ways in which doulas' support of AA women can contribute to healthy, positive, birth experience.

## Methods

We recruited doulas with a range of years of experience, with a special emphasis on doulas who work with low-income or AA women (Table 1). A combination of purposive and snowball sampling was used to recruit doulas for participation. The majority of doulas were recruited through a nonprofit, The Birth Circle (TBC). TBC is a community and birth doula organization in the greater Pittsburgh area that provides services for free or at low cost, specifically to women who are members of the local medical assistance product, University of Pittsburgh Medical Center Medicaid for You Program.^24^ Seven doulas from TBC participated in the study, and three additional doulas, referred by participants or local doulas and working outside of TBC, were recruited to participate. The final sample for this study was 10 doulas. Doulas are referred to by letters A–J in this article to maintain confidentiality. All participants provided verbal consent and were compensated $30 for their participation. No identifying information was collected from participants. This project was exempt by the University of Pittsburgh Institutional Review Board.

**Table 1.** Demographic Characteristics of 10 Participating Doulas

Characteristic	*n* (%)
Race ethnicity	
Black or AA	5 (50)
Hispanic or Latino	1 (10)
Asian or Pacific Islander	1 (10)
Bi-racial	1 (10)
White	2 (20)
Years of doula practice	
<5	4 (40)
5+	6 (60)
Career outside of being a doula	
Yes	8 (80)
No	2 (20)
Percentage of clients who are black or AA	
0–25%	1 (10)
26–50%	2 (30)
51–75%	3 (30)
76–100%	3 (30)
Percentage of clients who are of low income	
0–25%	0
26–50%	0
51–75%	1 (10)
76–100%	9 (90)

AA, African American.

The semistructured interview guide (Appendix A) and brief demographic questionnaire (Table 2) were developed based on literature on the potential benefits of doulas and four key-informant interviews with local home-visiting and doula program administrators. In-depth interviews were designed to capture maximum information about the doulas' experiences working with AA and low-income mothers, with emphasis on the doulas' perceptions of persons' experience, specific services offered, and challenges of providing services. Interviews were recorded, transcribed verbatim, and analyzed using grounded theory methods.^25^ We determined codes initially through line-by-line *in vivo* coding of interviews by two researchers who coded independently, and then secondarily by researchers comparing codes. Where there was any disagreement in interpretation, transcript portions were reread for context, and codes were discussed until consensus was reached. The codebook was refined after coding of each transcript to reflect new information. In a third wave of analysis, codes were displayed, organized into overarching themes, and the relationships of these themes to one another was determined. This was carried out with special attention to concepts that emerged around the methods doulas use to support mothers who are AA, have low income, or both.

**Table 2.** Demographic Questionnaire

This is a brief questionnaire to capture information not addressed in the interviews. This will be aggregate data for the group. This information is confidential and will not be linked to your interview.	
1. How long have you been in practice as a doula?	___________________
2. By your best estimate, what percentage of your clientele is African-American	• 0–25%
	• 26–50%
	• 51–75%
	• 76–100%
3. Do you have a career outside of being a doula?	• Yes, please describe: __________
	Is is:
	○ Full-time
	○ Part-time
	• No
4. By your best estimate, what percentage of your clientele is “low-income”?	• 0–25%
	• 26–50%
	• 51–75%
	• 76–100%
5. Which doula service/company/organization are you affiliated with? (Please select all that apply)	• The Birth Circle
	• Birth Doulas of Pittsburgh
	• Pittsburgh Doula Network
	• Independent
	• Other: __________________
6. How does most of your clientele typically find you? (Please select all that apply)	• Word of mouth
	• Social media (Facebook, Twitter, etc.)
	• Doula certifier (DONA.com, childbirthinternational.org, prodoula.com, cappa.net)
	• Referral from: ________________________
	• Other: __________________
7. You identify as:	• Black or African American
	• Hispanic or Latino
	• Native American or American Indian
	• Asian/Pacific Islander
	• Bi-racial
	• Other: __________________

## Results

Ten doulas were interviewed who had years of experience ranging from 2 to 14 years, with an average of 6.4 years of practice. All doulas had experience working with birthing persons who are AA, have low income, or both: 60% of doulas reported having a client base of 50% or more AA, and 100% of doulas reported a client base that is 50% or more with low income from their perspective. Eighty percent of doulas were affiliated in some way with TBC and 20% worked as independent or private doulas. Fifty percent of the doulas identified as AA or black, 20% white, and one doula representing each of the following racial/ethnic groups: Hispanic or Latino, Asian or Pacific Islander, and bi-racial. The demographic characteristics of the sample are included in Table 1.

In interviews, doulas shared the ways they perceive themselves as centering birthing individuals, specifically low-income AA women. In this helping these individuals feel empowered in a system that often works to disempower women of color and of lower socioeconomic status (SES). Three major themes emerged, highlighting the experience of doulas (Table 3): (1) similarities of race, culture, and experience impact care; (2) doulas provide birthing persons with support and resources beyond birth, and (3) doulas recognize the institutional biases that exist in the health care system and try to mediate their effect on birthing persons.

**Table 3.** Overview of Emergent Themes

Theme	Description
1. Similarities of race, culture, and experience impact care	Racial, cultural, and experiential similarities between mother can help in the creation of a trusting mother–doula relationship
2. Doulas often step outside their role to provide mothers with extra support but cannot do it all	Doulas often link mothers to outside resources, such as nutrition and housing supports. At times find themselves as the provider of resources, such as clothing or rides to medical providers; however, taking on the extra tasks and services can lead to burnout and doulas feeling overextended
3. Doulas recognize the institutional biases that exist in the hospital system	Doula help prevent overt effects of institutional racism, such as ensuring mothers are asked consent of procedures and are addressed respectfully by medical staff

**Appendix A.** Interview Guide

Interview guide	
Background	Why did you decide to become a doula?
	How do you see your role as a doula?
	Probe: Do you see yourself as a community or birth doula?
	Probe: How do you identify yourself as a doula?
Characterization of mother's experience	What do mothers think you do?
	Probe: How much do mothers know about what you do?
	Probe: How do mothers learn about you as doula?
	What are some of the most important things you do to support mothers?
	Probe: Tell me about the points of pregnancy, delivery, and postnatal where you provide support?
Specific services and their utilization among marginalized mothers	Tell me about the other services you offer to mothers as a doula
	Probe: Are any of the tasks you perform more time-intensive? Tell me about them.
	Probe: Are any of the tasks you perform more costly? Tell me about them.
	In your experience, what services do mothers tell you they find most useful?
	Probe: What services do mothers request the most?
	Probe: Do you find that low-income mothers request certain services more than other services? Tell me about these.
	Probe: Are there services that you find AA mothers request the most? Tell me about these.
	Probe: What do you think is the biggest challenge for these women?
Challenges of providing service	What are some of the challenges you face when providing services?
	Probe: Are any tasks especially physical or emotionally difficult? Tell me about those.
	Probe: Have you had experiences where your race/ethnicity has influenced your experience with a mother? Tell me about those. You can tell me about positive or negative experiences.
	Probe: Tell me about any differences when dealing with mothers who are your same race or mothers who are different race.
Characterization of training	What part of your training has helped you the most?
	Are there things you wish you had more training in or exposure to, to prepare you for your work with women who are AA and/or with low income?
	Probe: What do you think would improve doula services in Pittsburgh?
	Probe: What do you think would improve home-visiting services for you?
Closing	Is there anything else you would like to share with me?

AA, African American.

### The influence of similarities of race, culture, and experience

The first emergent theme is the influence of similarities of race, culture, and experience, and its impact of the relationship between birthing person and doula. Inherently, a doula's role is to provide support to birthing individuals; however, the ability to accept this support is influenced by the person's ability to trust the doula. In interviews, doulas shared the significance of race and culture when working with people who are often mistreated by society and consequently the health care system, one such group being AA women. Because of this treatment, AA women may develop feelings of mistrust toward the health care system. This mistrust can manifest itself in a way where AA mothers feel uncomfortable working with white doulas, feelings of mistrust that may not be present if working with a doula with the shared experience of being AA. This idea is captured by Doula B:

I know that some of my [black] clients are fearful. “What if I get a white doula?” I always say, “You're never going to meet a mean doula. It doesn't matter what color they are” …But a lot of the African American women are afraid… I had one just flat-out say to me on the phone, “I don't want a doula.” And I said, “Okay, that's fine, can I ask you why?” She says, “I don't want you coming in here telling me what I need to do, how I need to do it.” And I said, “Okay. Well, what makes you think I would do that?” “All you white people are like that” I said, “Well, I'm not white…Do you want a doula now?” She said, “Prove it.” So I Facetimed her.

At times, racial concordance was not enough to help foster a trusting relationship between birthing person and doula. In these instances, doulas shared that a similarity in life or cultural experience helped foster a trusting relationship where clients could feel comfortable receiving doulas' support. This was captured in the experience of Doula D:

Sometimes I feel like some moms are not comfortable with me… And I feel like if a woman feels more comfortable with somebody of their own skin color, that she should have that. There's times where it doesn't make a difference at all, and you can just connect with a person on a different level, like both being single moms. [I want moms] to feel comfortable with me, and maybe it's not her, maybe she's never been treated right by somebody who's white, then I might not be a safe person for her… It's not just race…sometimes there's socioeconomic or education level. I've had white moms reject me too because I don't fit into their pattern of comfortable…if I can find that point of relation, and you can help people to see how we are alike in some way and we can connect, it works usually. But there's times where that woman probably sees me and she's like, “This person is nothing like me, and I can't relate to her, and she's not going to make a difference for me.”

Finding a point of similarity between birthing person and doula helps build a trusting relationship between the pair. This point of similarity can be race; however, it can also be a shared life experience as indicated by Doula D.

### Doulas' work beyond assisting with birth

The second emergent theme is how doulas often provide additional supports, and link clients to other resources. Participating doulas were community doulas, and thus already provided more help to birthing persons outside the traditional birth roles. Doula G shared that when working with an individual who may need more help, she will link them to:

WIC for example, Nurse-Family Partnerships, Healthy Start. These are all organizations that provide support to mothers free of charge. We might want to remind her the importance of getting a library card…we will connect her with organizations that will provide either Uber service at no charge to her, or she would get a ConnectCard…We want to connect her with some of those resources in the community.

Many doulas shared how they found themselves providing skills and resources outside of what is typically provided by a birth or community doula. One doula shared she would provide transportation for mothers to their prenatal visits, another that she would often go through her own children's clothing to find items to give to mothers she worked with, both activities neither required nor necessarily recommend of their service. In addition, doulas shared they needed to learn that they could not do everything for a mother. They spoke about the high risk of burnout and the potential to overextend oneself. Doula D shared her realization of this challenge:

…The biggest challenge I had to learn in doula work is what my scope is as a doula…learning that I don't have to solve all the problems in her life, that's not my job, even though part of me wants to. That I'm there to do doula services. I can help prepare her for her birth, and I can help her transition to motherhood afterward. That's what I can do. And I can refer to other people for other things.

Despite the best intentions, attempting to help a client in every way is not feasible, nor does it always work. Doulas spoke of the difficulty that arose when it was time to end their relationship with clients, as some became dependent on the doulas' services. Other doulas shared experiences of linking clients to resources, when at times systems were set up in ways that clients could not benefit. For example, overextended public housing programs unable to provide housing or clients who once connected with a resource did not use it.

### Doulas recognize and navigate institutional biases in the hospital system

The final theme that emerged is how doulas recognize and navigate institutional biases in health care system. Doulas shared experiences of witnessing the mistreatment of the birthing persons they worked with by hospital staff, including a client not being asked consent for a pelvic examination, and hospital staff's use of disparaging language to talk to laboring person, for example making derogatory comments on the number of children a woman has. In these instances, a doula may feel it appropriate to step in or mediate situations. Doula G provides an example of how she does this:

Whereas this black momma because her socioeconomic status looks different, she's not receiving the same treatment. I've seen that dozens of times where the doctor comes in, shoves her leg open, and does a pelvic exam without even talking with the mother…this isn't something we're just making up. We're seeing this where black women and women of color and immigrant women are not receiving the same treatment, and that we can't just stand by and watch this. We want to teach our moms how to speak up for themselves. And sometimes that means we have to stand in the gap and say, “Could you wait?” even if [mother] can't…And they might get mad and huff and puff and leave the room, but if they decide to examine that woman anyway, that provider needs to be reported.

Doula G helps clients recognize they are being treated unfairly, and then when possible, helps clients be the ones to speak in their own defense. For example, suggesting to a change of doctors, or holding nurses accountable for their language.

At times, a doula must use her own voice, as someone with experience attending multiple births, to speak on behalf of the client when they witness inappropriate treatment they believe will have a negative physical or emotional impact on a laboring person. However, doulas have a philosophy to “never argue over a laboring woman's body,” a philosophy that can at times be difficult to uphold. AA doulas shared how they would witness subtle mistreatments that AA clients would not perceive. Doula J shares how she navigates these scenarios:

I have to continue to be action-oriented. How can we navigate this issue or how can we move forward? You have to kind of just stay focused. Because if [mom's] offended, it's easy for me to get offended, and then grandma gets offended, and now, we're all in here offended. And that's not helping anybody. You don't want to stress moms out because that then prolongs the labor. So you just really have to always be cognitive of how is mom feeling, let me not add any more pressure or stress to her. Sometimes that means biting your tongue.

Because the birthing person is the greatest priority, direct mediation is not always the best option. Doulas must balance knowing that a client is being treated differently while simultaneously remaining present to a laboring person's needs and feelings.

## Discussion

This study was an exploration of perspectives of community doulas working with a specific population of low-income AA women in a mid-size urban area. Because of documentation that captures the detail in these doulas' narratives, and the rigorous, iterative process of analysis used by the researchers, we are confident that we have presented findings grounded in the lived experiences of a group of community doulas. These findings contribute to a small but growing body of evidence of the importance of doulas.

The first theme that arose from interviews was that similarities of race, culture, and lived experience impact care. Although there is currently no research on the race concordance of doulas and their clients, similar research shows that race similarity between physicians and patients influences patient's comfort with their provider.^26,27^ For example, some AA patients tend to believe that AA physicians will better understand their health.^26,27^ The discomfort some AA women may experience may be because of historical mistreatment (such as enslaved women being used as test subjects to pave the way for modern gynecology) and current mistreatment at the hands of medical practitioners, for example, inadequate pain relief for black patients because of physicians' false belief that black patients have higher pain tolerance, and negative word-of-mouth experiences from friends and family.^28–33^ However, in this study, doulas shared that at times, race was not enough of a similarity to gain trust from clients. Understanding the sordid racial history helped doulas understand their clients' fears, and look to other shared experiences, like faith or lived experience, to help build relationships. Awareness of this distrust may also lead persons of color to enter the doula profession. A recent study on motivations of women of color becoming doulas found that many women became doulas so they could provide culturally competent care in their communities.^4^

We also found that community doulas often provide birthing persons with supports beyond pregnancy and delivery, with positive results, as has been shown in other research.^3,4,8,10,34–36^ For example, home visiting, breast-feeding assistance, mentoring, and involving partners.^3,10,11,35,37^ However, this study highlights the stress some doulas face, when these services are still not enough for the birthing persons they serve. In literature, the relationship between witnessing mistreatment of laboring women and burnout of birth doulas has been observed; however, research has not explored how overextending oneself might influence burnout among community doulas.^38^

The final theme that emerged from interviews was that doulas recognize the institutional biases that exist in the health care system and try to mediate their effect on birthing persons. According to literature, many low-income, AA women face social disadvantages because of their race, sex, and SES, with the intersection of multiple sources of oppression often negatively impacting health.^31,38^ Women who endure the daily stress of being both AA and poor, may also endure stress from various other negative social circumstances, such as housing and food insecurity that disproportionately affect low income and AA women.^39,40^ However, new evidence shows doulas, in providing emotional, physical, and tangible support, can help reduce the negative effects of these stressors on birthing persons.^4^

These themes highlight only a subset of the complex dynamics that impact doulas and their work. These community doulas manage the challenges and stressors of supporting low-income AA women in birth: working to build trust with them, buffering inappropriate behavior by medical personnel, supporting and advocating for their clients, and attempting to balance meeting the multitude of needs of their clients while avoiding their own burnout. Some of these dynamics may be particular to, or more notable when, working with low income and AA women. There is potential to gain a further understanding of the ways in which doulas support birthing persons and promote positive outcomes.

Given the growing body of evidence of the positive influence that doulas can have on birth experiences and outcomes, the experiences and perspectives of doulas working with vulnerable populations may help to inform systems that utilize their services. In particular, community doulas, as those described in this study, may play a critical role in helping to reduce health inequities experienced by low-income AA women, and as such deserve consideration.

### Strengths and limitations

The primary limitation of this study is that, despite efforts for more sampling breadth, the majority of the doulas worked at a nonprofit community doula organization, and no private doulas were included in this study. In addition, doulas were asked to share their experience with women, who to the doula's knowledge, identified as low-income and AA. Future work that explores similarities and differences in the experiences and perspectives of doulas depending on a variety of employment, such as community-based doulas not associated with a nonprofit organization, or private doulas, may have different findings.

## Conclusion

Doulas' support and services should not be relegated as a privilege only some persons are able to experience. Further exploration is needed centering the experience of birthing persons who are not white nor of higher SES. Future work should focus on exploring the clinical impact of community doulas on various racial and socioeconomic populations, reducing barriers to access, and how to communicate doula benefits, with an emphasis on doulas work to mediate the effects of institutional biases.

## Abbreviations Used

AA: African American
SES: socioeconomic status
TBC: The Birth Circle
